# The effectiveness and safety of Yi Guan Jian decoction in the treatment of primary liver cancer: A systematic review and meta-analysis of randomized controlled trials

**DOI:** 10.1097/MD.0000000000047153

**Published:** 2026-01-09

**Authors:** Zhilin He, Xindan Cao, Li Zhao, Shidong He

**Affiliations:** a School of Chinese Medicine, Faculty of Medicine, The University of Hong Kong, Hong Kong, China; b Department of Traditional Chinese Medicine, Dongguan Maternal and Child Health Hospital, Dongguan, Guangdong, China; c Department of Oncology, Dongguan Hospital, Guangzhou University of Chinese Medicine, Dongguan, Guangdong, China.

**Keywords:** meta-analysis, primary liver cancer, traditional Chinese medicine treatment, Yi Guan Jian decoction

## Abstract

**Background:**

Yi Guan Jian decoction (YGJ) is a well-known traditional Chinese medicine (TCM) formula that has been widely used in the clinical treatment of primary liver cancer (PLC). The primary objective of this study is to systematically evaluate the clinical efficacy and safety of YGJ decoction in the treatment of PLC.

**Methods:**

We searched China National Knowledge Infrastructure, Wanfang Data, Chinese Science and Technology Journal Database (VIP), PubMed, Sinomed, Web of Science, and Cochrane Library from inception to March 2025. Randomized controlled trials involving YGJ or its modified formulations for PLC treatment were included. Study quality was assessed using RevMan 5.4. Count data were analyzed using relative risk and odds ratio; continuous data were analyzed using mean difference (MD) and standardized MD. All statistical indicators were calculated with 95% confidence intervals (CI) and *P* values. Data were grouped and analyzed based on intervention duration and control group treatment methods.

**Results:**

A total of 10 randomized controlled trials were included, involving 745 patients. Meta-analysis showed that YGJ or its modified versions, combined with Western medical supportive treatments, significantly increased treatment effectiveness (OR = 1.84, 95% CI: 1.32–2.58), improved Karnofsky performance status score (MD = 7.00, 95% CI: 4.80–9.21), decreased serum alpha-fetoprotein (AFP) (SMD = −0.36, 95% CI: −0.58 to −0.14), improved TCM syndrome scores (MD = −3.10, 95% CI: −3.78 to −2.42), reduced total bilirubin (MD = −1.52, 95% CI: −2.25 to −0.78), and lowered gastrointestinal adverse reactions (OR = 0.53, 95% CI: 0.33–0.84).

**Conclusion:**

The combination of YGJ or its modified versions with modern medical therapies significantly enhances treatment effectiveness, Karnofsky performance status scores, and TCM syndrome scores, reduces AFP levels, and decreases gastrointestinal adverse reactions compared to modern medical therapies alone. These results suggest that YGJ may be a valuable addition to clinical treatment strategies. However, the included studies had small sample sizes, were geographically limited, and mostly lacked blinding. Future research should involve larger, more diverse samples and rigorous study designs to confirm these findings.

## 1. Introduction

Primary liver cancer (PLC) is a malignant tumor that originates from hepatocytes and intrahepatic biliary epithelial cells.^[1]^ In its early stages, PLC typically presents without obvious symptoms. As the disease progresses, patients may gradually develop clinical manifestations such as liver pain, fatigue, anorexia, and weight loss.^[2]^ The treatment of PLC often involves a multidisciplinary approach with various therapeutic modalities, including hepatectomy, liver transplantation, ablation therapy, endovascular interventional therapy, radiotherapy, systemic antitumor therapy, and traditional Chinese medicine (TCM). Each modality has its own advantages and disadvantages, with some overlap in indications.^[3,4]^ However, these treatments often face limitations in efficacy, including high recurrence rates, drug resistance, severe adverse reactions, and difficulty in significantly extending patient survival.^[5,6]^ For early-stage PLC, surgical resection is generally the primary treatment; however, the 5-year recurrence rate after radical resection can reach 62%.^[5]^ Despite increasing public health awareness and the popularization of early detection and screening, 50% to 70% of PLC patients still present with advanced disease at diagnosis, losing the opportunity for surgical intervention.^[7]^ This highlights the need for improved early detection and more effective therapeutic strategies to address the challenges posed by PLC.

In TCM theory, PLC is pathologically classified under disease categories such as “abdominal mass” (Jī Jū), “accumulation syndrome” (Jī Jí), and “tympanites” (Gǔ Zhàng), characterized by core pathogenesis involving liver-kidney yin deficiency. Contemporary clinical observations have further validated that yin deficiency of the liver and kidney constitutes both the fundamental pathogenesis and predominant clinical manifestation in PLC progression.^[8]^ Aligned with the TCM therapeutic principle of “fortifying healthy qi while eliminating pathogenic factors,” integrated treatment regimens combining modern medical technologies with TCM-based individualized interventions have demonstrated unique clinical advantages. These synergistic approaches show particular efficacy in mitigating radiotherapy/ chemotherapy-induced adverse effects, modulating tumor microenvironment homeostasis, enhancing immune surveillance function, and reducing postoperative recurrence rates.^[9]^ As a classical formula for nourishing yin and dispersing liver stagnation, Yi Guan Jian decoction (YGJ) has shown promising therapeutic outcomes in PLC management through multidimensional mechanisms including symptom palliation, quality of life improvement, and potential survival prolongation.^[10]^

The liver and gallbladder are interconnected in TCM, with the liver’s free flow function strongly related to the gallbladder’s excretion function. Bilirubin, a key component of bile, represents the liver’s metabolic and excretory processes. Elevated bilirubin levels often suggest compromised liver function, which is common in PLC patients. Monitoring bilirubin levels allows for an indirect assessment of YGJ’s effect on liver metabolism and excretion.^[11]^ Serum alpha-fetoprotein (AFP) is an embryonic protein that is very slightly expressed in adult livers and acts as a particular biomarker for PLC, with levels correlated to tumor growth or therapy response.^[12]^ In TCM, liver cancer growth is connected to liver malfunction, qi stagnation, and blood stasis. Assessing AFP levels helps evaluate YGJ’s impact on tumor load. Incorporating these clinical markers into TCM evaluations is crucial for a comprehensive assessment of interventions like YGJ. By clearly linking TCM theory with clinical outcomes such as bilirubin and AFP, we can enhance the scientific rigor and credibility of TCM research in PLC management.

Despite these unique advantages, YGJ shares similarities with other TCM treatments in certain aspects. A meta-analysis of the treatment of primary hepatocellular carcinoma with different radiotherapy regimens using Compound Cantharidin Capsules showed that the drug combined with Transarterial chemoembolization (TACE) intervention could effectively improve the clinical symptoms of patients and could significantly reduce the incidence of myelosuppression.^[13]^ Another Meta-analysis that included several articles on the treatment of primary hepatocellular carcinoma with TCM showed that the combination of TCM with Western medicine was superior to the treatment with Western medicine alone in terms of improving the near-term efficacy of the tumor, improving the AFP, liver function (aspartate aminotransferase, alanine aminotransferase, total bilirubin), immune indexes, and decreasing the adverse effects.^[14]^ However, to date, no studies have directly compared the therapeutic efficacy of YGJ with other herbal formulations in the treatment of PLC, highlighting a gap in the literature that warrants further investigation.

Based on this background, this study systematically collected clinical experimental data on YGJ treatment for PLC. The aim was to objectively analyze its efficacy and provide valuable insights for the clinical application of TCM in PLC management.

## 2. Materials and methods

The preferred reporting items for systematic reviews and meta-analyses (PRISMA 2020) declaration was adhered to in this systematic review.^[15]^ The PROSPERO registration number for this systematic review is CRD420250651467.

### 2.1. Inclusion criteria

Study type: Randomized controlled trials (RCTs) involving YGJ or modified YGJ for the treatment of PLC, without language restrictions; Study subjects: All patients met the diagnostic criteria for PLC established in the Clinical Practice Guidelines: Oncology; The treatment group used a combination of TCM and Western medicine, while the control group used Western medicine alone; Outcome measures: Total effective rate, Karnofsky performance status (KPS) score, AFP, total bilirubin, TCM syndrome score, and adverse reactions (gastrointestinal reactions, bone marrow suppression).

### 2.2. Exclusion criteria

Review articles, experience reports, and exploratory studies; articles with dubious data; articles lacking the aforementioned outcome measures; animal experiments, pathway studies, and other nonclinical RCT articles; clinical studies combining YGJ with other formulas.

### 2.3. Search strategy

We searched the following databases for clinical RCTs on the treatment of PLC with YGJ or modified YGJ: China National Knowledge Infrastructure, Wanfang Data, Chinese Science and Technology Journal Database (VIP), PubMed, Sinomed, Web of Science, and Cochrane Library. The search period covered from the inception of each database to March 16, 2025. We additionally obtained completed yet unpublished studies from clinicaltrials.gov and monitored the outcomes of these studies. This study only included peer-reviewed full-text articles. Conference abstracts, unpublished studies, and gray literature were not included to ensure the quality of the studies and the integrity of the data. Only studies published in Chinese or English were included. A meticulously designed search strategy was employed to ensure comprehensive data retrieval. The search formula utilized in PubMed is as follows: (yiguanjian decoction [Supplementary Concept] OR Yi Guan Jian OR Yi guanjian OR Modified Yiguanjian OR Yiguanjian Plus) AND (PLC OR Liver Cancer OR Hepatic Tumor). The search strategies for all databases are detailed in Appendix 1, Supplemental Digital Content, https://links.lww.com/MD/R124.

### 2.4. Literature screening and data extraction

Two researchers (Zhilin He and Xindan Cao) independently screened the literature and extracted data, then merged and cross-checked the results. In case of disputes over the inclusion of certain articles, a third researcher (Shidong He) was consulted. The extracted information included: basic literature information (title, author, publication year), experimental basic information (patient age, treatment duration, dosage and administration of TCM, Western medical treatments, whether blinding was used, whether random allocation was performed, etc), and outcome measures (total effective rate, KPS score, serum AFP, TCM syndrome score, gastrointestinal reactions, bone marrow suppression, etc).

### 2.5. Literature quality assessment

Based on the quality assessment criteria provided by RevMan 5.4, the included studies were evaluated for bias risk using 7 indicators: random sequence generation, allocation concealment, blinding of participants and personnel, blinding of outcome assessment, incomplete outcome data, selective reporting, and other biases. The evaluation levels were categorized as low risk, unclear, and high risk.

### 2.6. Statistical methods

The data analysis was performed using RevMan 5.4 software. For count data, relative risk and odds ratio (OR) were employed, whereas for continuous data, mean difference (MD) and standardized mean difference (SMD) were utilized. All statistical indicators were calculated with 95% confidence intervals (CI) and *P* values. If *P ≥ *.1 or I^2^ > 50%, the sources of heterogeneity were explored through subgroup analysis or discussion, and a random-effects model was used instead. Conversely, a fixed-effect model was applied. A *P* value of ≤ .05 indicated a statistically significant difference among the data.

## 3. Results

### 3.1. Literature search results

**Figure 1.**
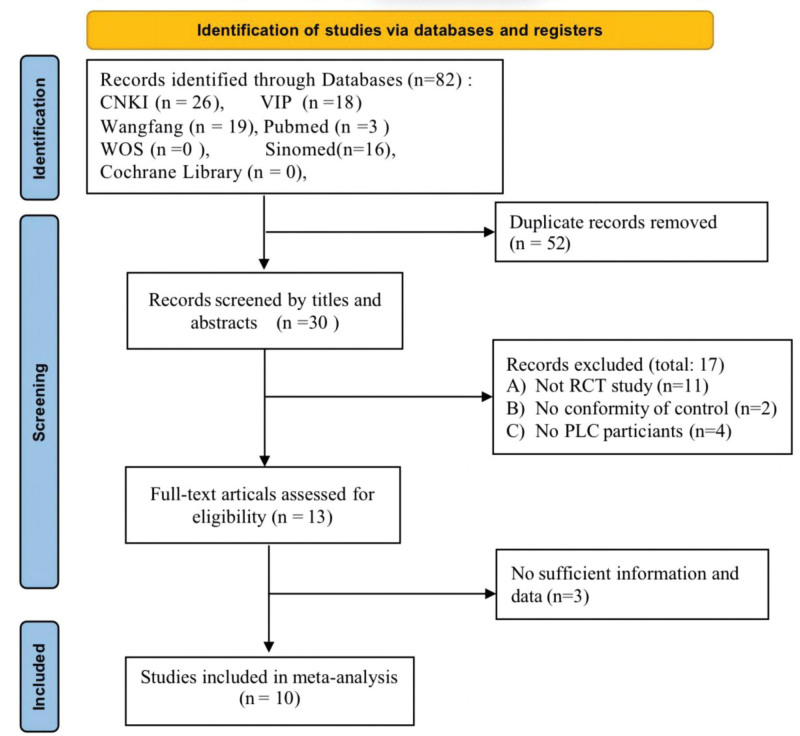
Flowchart of study selection.

Initially, a total of 82 articles were identified through the search strategy. Following the removal of 52 duplicates, 30 articles remained. Subsequently, 17 articles were excluded based on the exclusion criteria after screening the titles and abstracts, leaving 13 articles. After reviewing the full texts of the remaining articles and comparing the data and conclusions within, 3 articles with dubious or missing data were excluded. Ultimately, 10 articles^[16–25]^ were included in the analysis. A flowchart of the literature screening process is illustrated in Figure 1.

**Figure 2.**
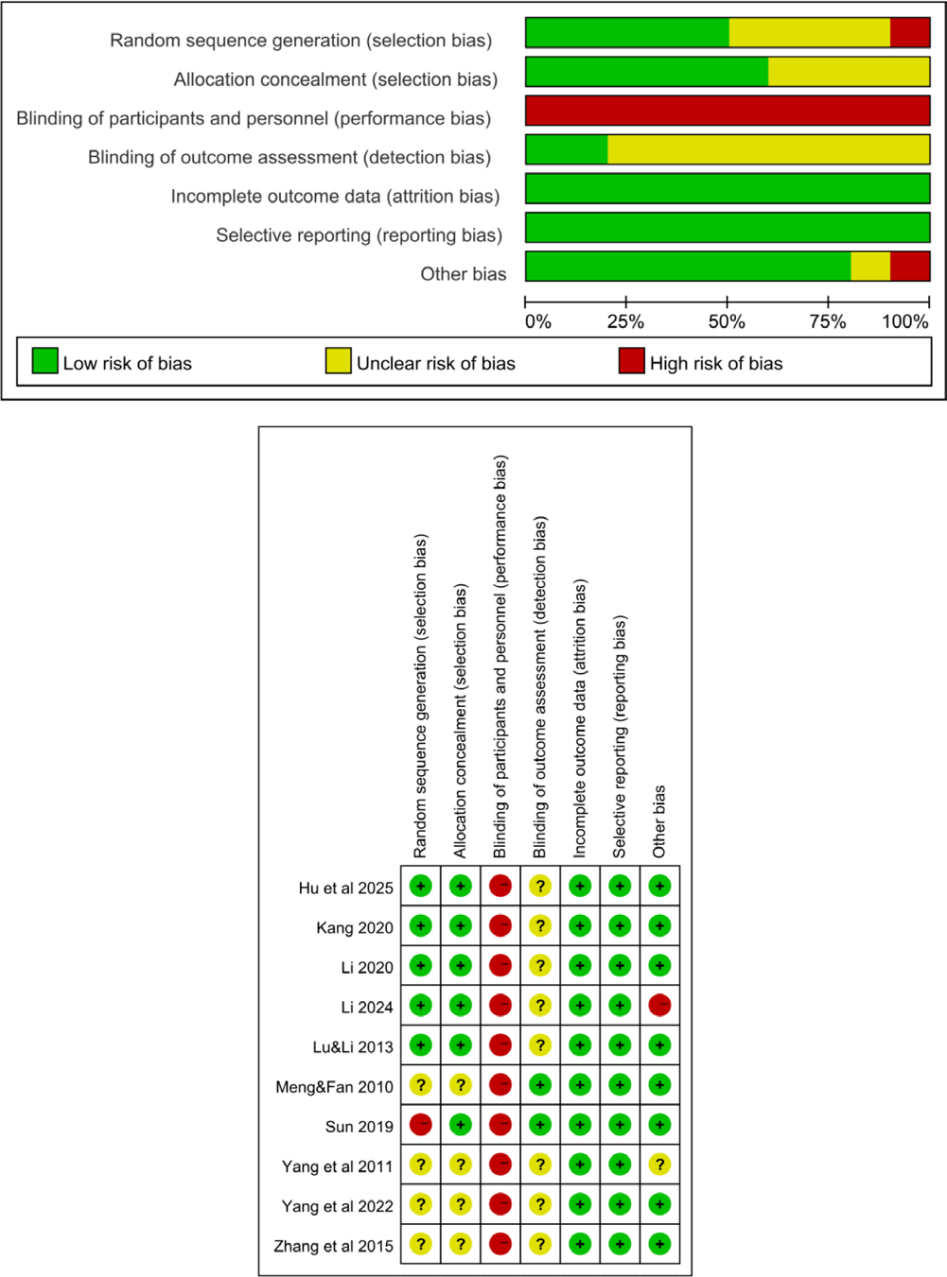
Risk of bias graph.

In this review, a total of ten RCTs were included. Among the ten included studies, regarding randomization, 5 studies used randomization methods, 4 studies did not explicitly state their methods, and 1 study did not use randomization, presenting a high risk of bias. For allocation concealment, 6 studies implemented effective measures, while 4 studies did not report clearly. None of the studies achieved double blinding, resulting in a high risk of performance bias. In terms of data integrity, data from all 10 studies were complete. Regarding selective reporting, no selective reporting was found in the 10 studies. For other biases, 8 studies showed no other biases, 1 study was unclear, and 1 study identified other biases. Additionally, 2 studies showed no blinding of outcome assessment, and 8 studies were unclear. The quality assessment table for the included studies is illustrated in Figure 2, and the basic information of the included studies is detailed in Tables 1 and 2.

**Table 1:** Basic characteristics of the included studies (part 1).

Study	Number (E/C)	Age (year)		Male/female		Interventions	Comparators	Duration	Outcomes
		E	C	E	C				
Sun 2019^[16]^	30/30	65.69 ± 1.38	65.53 ± 1.25	17/13	16/14	YGJ + oral apatinib mesylate	Oral apatinib mesylate	NR	①
Lu and Li 2013^[17]^	42/38	48.32 ± 16.23	49.65 ± 17.14	NR	NR	YGJ + TACE	TACE	2 mo	①②③⑤
Zhang et al 2015^[18]^	51/51	49.30 ± 6.62	51.80 ± 7.12	35/16	37/14	YGJ + argon-helium knife	Argon-helium knife	4 wk	①②
Li 2020^[19]^	30/30	54.03 ± 10.07	53.10 ± 8.78	22/8	23/7	YGJ + gamma knife	Gamma knife	2 mo	①②③④⑥⑦
Li 2024^[20]^	32/30	54.75 ± 8.03	56.30 ± 8.93	22/10	19/11	YGJ + oral lenvatinib mesylate	Oral lenvatinib mesylate	2 mo	①②③④⑥⑦
Meng and Fan 2010^[21]^	40/40	40–78		NR	NR	YGJ + IL-2 intravenous injection	IL-2 intravenous injection	6 wk	①
Yang et al 2022^[22]^	34/34	58.47 ± 11.77	59.13 ± 10.93	25/9	27/7	YGJ + TACE	TACE	6 mo	②⑥⑦
Yang et al 2011^[23]^	44/49	58.61 ± 11.33	56.31 ± 9.61	36/8	43/6	YGJ + basic Western medicine	Basic Western medicine	6 mo	②④
Kang 2020^[24]^	60/60	52.87 ± 10.14	53.67 ± 10.24	52/8	54/6	YGJ + TACE	TACE	4 wk	①②③④⑤⑥⑦
Hu et al 2025^[25]^	40/40	57.16 ± 9.15	58.69 ± 9.07	26/14	24/16	YGJ + Oral sorafenib mesylate tablets	Oral Sorafenib mesylate tablets	4 wk	①②⑤⑥

①Effective rate; ②KPS score ; ③Alpha-fetoprotein; ④Traditional Chinese medicine syndrome; ⑤Total bilirubin; ⑥Gastronintinal adverse reactions; ⑦Myelosuppression.

In this study, there were no significant differences (*P *> .05) between the treatment group and the control group in key characteristics such as age and gender at baseline, indicating good comparability between the 2 groups at baseline.

C = control group, E = experimental group, KPS = Karnofsky performance status, NR = not reported, IL-2 = interleukin-2, m = months, TACE = transarterial chemoembolization, w = weeks; outcome indicators.

**Table 2:** **Basic characteristics of the included studies (part 2**).

Study	Diagnostic criteria	Comorbidities
Sun 2019^[16]^	Unclear	None
Lu and Li 2013^[17]^	Clinical Diagnosis and Treatment Guidelines (Chinese Medical Association. 2005 Edition)	None
Zhang et al 2015^[18]^	Ministry of Health of the People’s Republic of China. Diagnosis and Treatment Standards for Primary Liver Cancer (2011 Edition)	None
Li 2020^[19]^	Ministry of Health of the People’s Republic of China. Diagnosis and Treatment Standards for Primary Liver Cancer (2017 Edition)	None
Li 2024^[20]^	Guidelines for Diagnosis and Treatment of Primary Liver Cancer (2022 Edition)	None
Meng and Fan 2010^[21]^	Unclear	None
Yang et al 2022^[22]^	Diagnosis and Treatment Standards for Primary Liver Cancer (2017 Edition)	None
Yang et al 2011^[23]^	The 2003 edition of the American Association of Oncology (AJCC) and the International Union Against Cancer (UICC) staging system for primary liver cancer, or the Barcelona staging system (BCLC) or the staging system of the Liver Cancer Professional Committee of the Chinese Anti Cancer Association	None
Kang 2020^[24]^	Diagnosis and Treatment Standards for Primary Liver Cancer (2017 Edition)	None
Hu et al 2025^[25]^	Guidelines for Diagnosis and Treatment of Primary Liver Cancer (2019 Edition)	None

### 3.2. Outcome measures

#### 3.2.1. Effective rate

**Figure 3.**
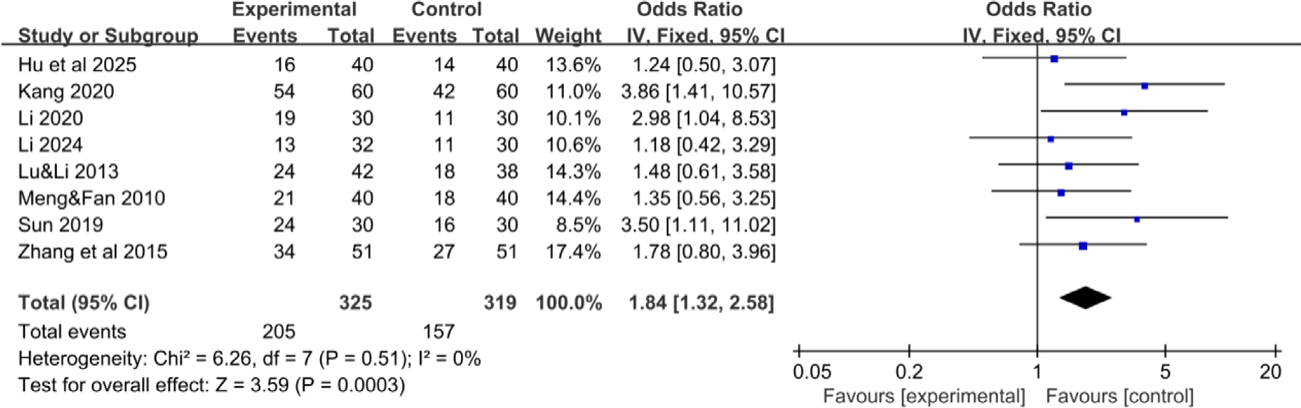
Forest plot of the pooled OR for treatment effectiveness and its 95% CI. CI = confidence interval, OR = odds ratio.

**Figure 4.**
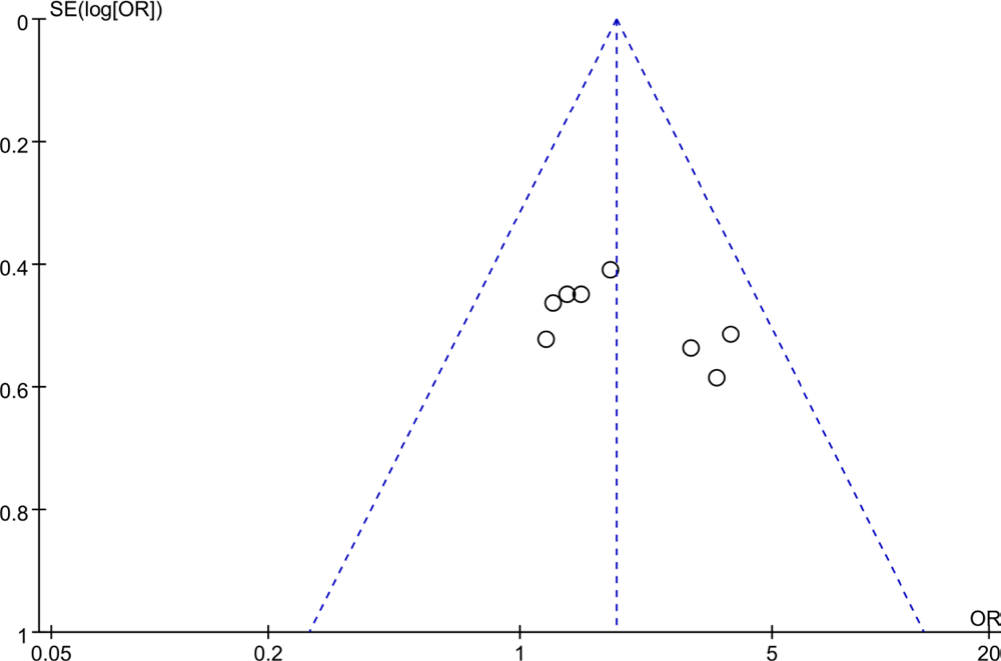
Funnel plot for effective rate.

In this meta-analysis, 8 RCTs^[16–21,24,25]^ reported data on the total effective rate of YGJ for PLC patients, encompassing 325 cases in the treatment group and 319 cases in the control group. As the heterogeneity of the pooled analysis results was low (I^2^ = 0%), indicative of low heterogeneity, thus justifying the use of a fixed-effect model for the meta-analysis. The difference was statistically significant (Fig. 3), indicates that patients treated with the combination of YGJ and Western medical treatments were 1.84 times more likely to achieve a higher total effective rate compared to those treated with Western medical treatments alone (OR = 1.84, 95% CI: 1.32–2.58, *P* = .0003). Given the substantial number of studies included and the potential for significant differences when employing imaging data as inclusion criteria, a funnel plot (Fig. 4) was constructed to detect any significant bias. As depicted in the figure, the studies were roughly symmetrically distributed on both sides and within the 95% confidence interval, with no conspicuous bias discernible.

#### 3.2.2. KPS score

**Figure 5.**
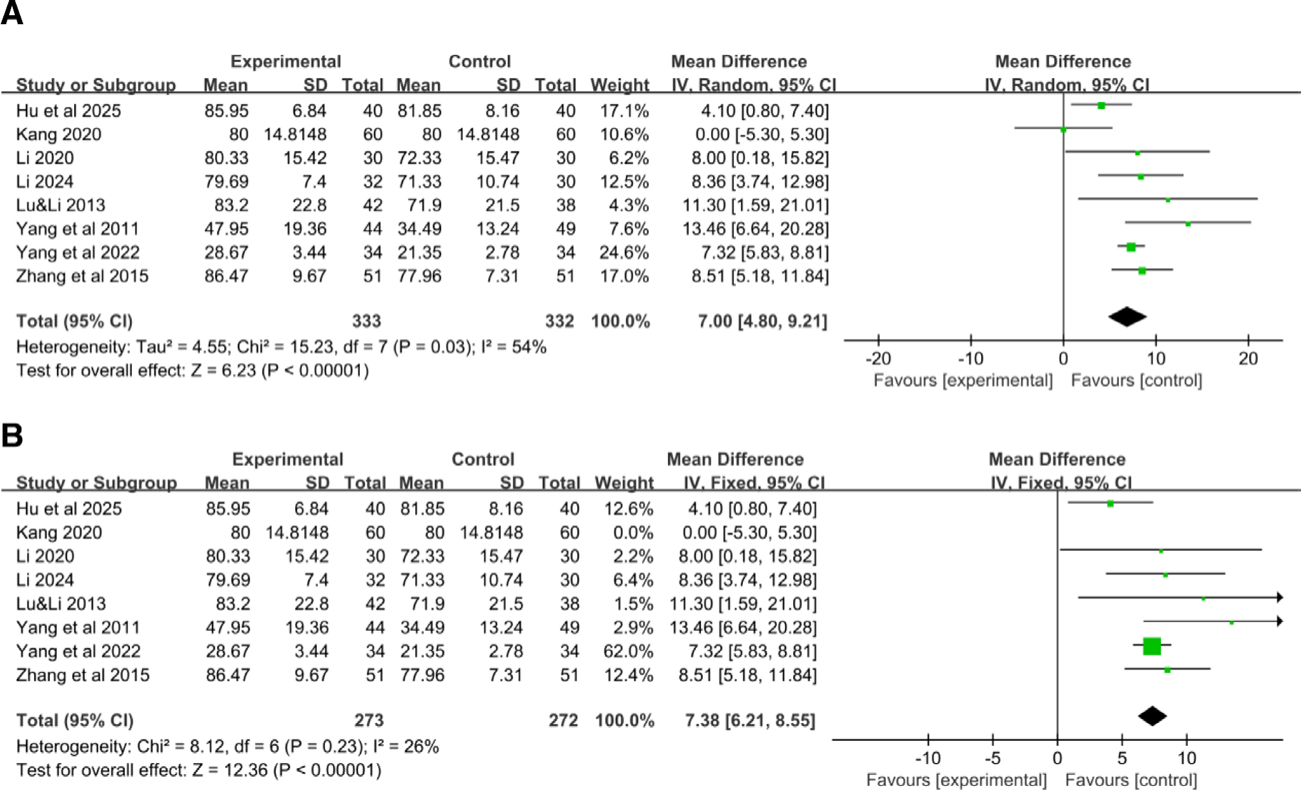
Forest plot of the pooled OR for KPS Score and its 95% CI. CI = confidence interval, KPS = Karnofsky performance status, OR = odds ratio.

Eight RCTs^[18–21,23–25]^ reported data on the impact of YGJ on the KPS score of PLC patients, with 333 cases in the treatment group and 332 cases in the control group. As shown in Figure 5A, the heterogeneity test indicated an I^2^ value of 54%, and thus a random-effects model was used. The result suggesting that, on average, patients treated with the combination of YGJ and Western medical treatments had a 7.00-point higher KPS score compared to those treated with Western medical treatments alone. This suggests a clinically significant improvement in the overall functional status and quality of life for patients with PLC when YGJ is used in conjunction with conventional therapies. (MD = 7.00, 95% CI: 4.80–9.21, *P* < .00001).

To identify the source of heterogeneity, a stepwise exclusion method was employed. It was found that after excluding Kang 2020, the heterogeneity decreased (I^2^ = 26%). Therefore, a fixed-effect model was used, considering that the high initial KPS score in Kang 2020 might be the source of heterogeneity (MD = 7.38, 95% CI: 6.21–8.55, *P* < .00001) (Fig. 5B).

#### 3.2.3. AFP

**Figure 6.**
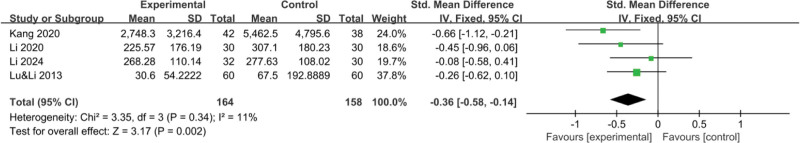
Forest plot of the pooled OR for AFP and its 95% CI. AFP = alpha-fetoprotein, CI = confidence interval, OR = odds ratio.

Four studies^[18,20,21,24]^ reported data on the impact of YGJ on serum AFP levels in PLC patients. The data from Kang 2020 were presented in quartiles and were converted to standard deviations for inclusion in this study, hence the use of SMD. A total of 164 cases were included in the treatment group and 158 cases in the control group. The heterogeneity test was low (I^2^ = 11%), and thus a fixed-effect model was used for the meta-analysis. The results showed that the serum AFP level in the treatment group was lower than that in the control group by 0.36 standard deviation units, which may reflect a positive impact on tumor burden or activity in patients with PLC (SMD = -0.36, 95% CI: −0.58 to −0.14, *P* = .002), with a statistically significant difference. The detailed results are shown in Figure 6.

#### 3.2.4. Traditional Chinese medicine syndrome score

**Figure 7.**
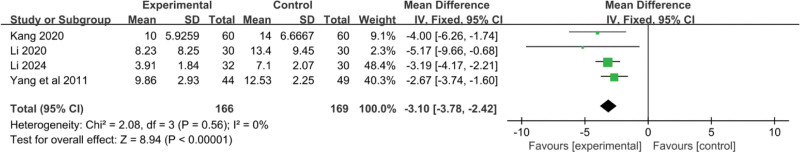
Forest plot of the pooled OR for traditional Chinese medicine (TCM) Syndrome Score and its 95% CI. CI = confidence interval, OR = odds ratio.

Four RCTs^[20,21,23,24]^ reported data related to TCM syndrome scores. A total of 166 cases were included in the treatment group and 169 cases in the control group. I^2^ = 0%, a fixed-effect model was used for the meta-analysis. The results showed that the treatment group had a more significant improvement in clinical symptoms compared to the control group (MD = −3.10, 95% CI: −3.78 to −2.42, *P* < .001), as shown in Figure 7.

#### 3.2.5. Total bilirubin

**Figure 8.**
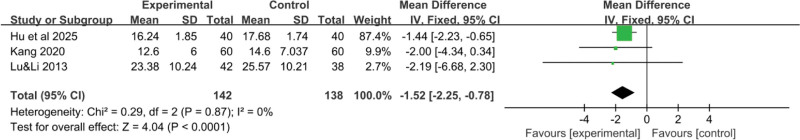
Forest plot of the pooled OR for total bilirubin levels and its 95% CI. CI = confidence interval, OR = odds ratio.

Three RCTs^[18,24,25]^ reported data related to total bilirubin levels. A total of 142 cases were included in the treatment group and 138 cases in the control group. I^2^ = 0%, a fixed-effect model was used for the meta-analysis. The results showed that the treatment group had a more significant reduction in bilirubin levels compared to the control group (MD = −1.52, 95% CI: −2.25 to −0.78, *P* < .0001), as shown in Figure 8.

#### 3.2.6. Gastrointestinal reactions

**Figure 9.**
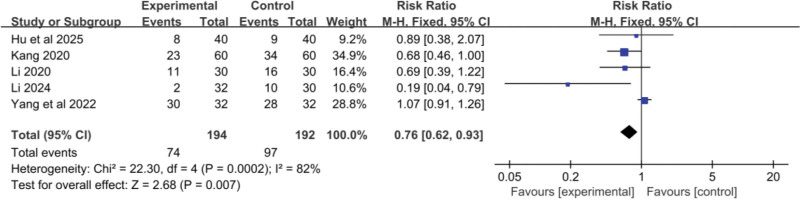
Forest plot of the pooled RR for gastrointestinal adverse reactions and its 95% CI. CI = confidence interval.

Five RCTs^[19,20,22,24,25]^ included data on gastrointestinal adverse reactions. A total of 194 cases were included in the treatment group and 192 cases in the control group. I^2^ =34%, a fixed-effect model was used for the meta-analysis. The meta-analysis results showed that the treatment group had a significantly lower incidence of gastrointestinal adverse reactions compared to the control group (RR = 0.76, 95% CI: 0.62–0.93, *P* = .007), with a statistically significant difference. See Figure 9.

#### 3.2.7. Bone marrow suppression

**Figure 10.**
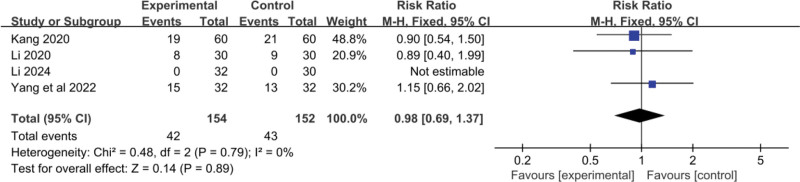
Forest plot the pooled RR for bone marrow suppression and its 95% CI. CI = confidence interval.

Four RCTs^[19,20,22,24]^ reported data on the impact of YGJ on bone marrow suppression in PLC patients. A total of 154 cases were included in the treatment group and 152 cases in the control group. As shown in Figure 10, I^2^ = 0%, and thus a fixed-effect model was used for comparison. The results showed no significant difference in the incidence of bone marrow suppression between the 2 groups (RR = 0.98, 95% CI: 0.69–1.37, *P* = .89). This suggests that YGJ may not have a significant impact on bone marrow suppression in PLC patients when compared to standard treatments.

### 3.3. Subgroup analysis

Due to the significant differences in treatment duration and Western medical treatment methods among the included studies, subgroup analyses were conducted, with total effective rate and KPS score as the main indicators. Guidelines indicate that PLC is typically evaluated for the first time 4 to 6 weeks after conventional treatments such as radiotherapy, immunotherapy, targeted therapy, and TACE.^[26]^ Therefore, this study uses 6 weeks as the node for subgroup analysis to observe the differences in the therapeutic effects of TCM interventions before and after 6 weeks. The intervention period was divided into 2 groups based on a 6-week threshold, and Western medical treatments were categorized into pharmacological and surgical groups.

#### 3.3.1. Total effective rate-treatment duration

**Figure 11.**
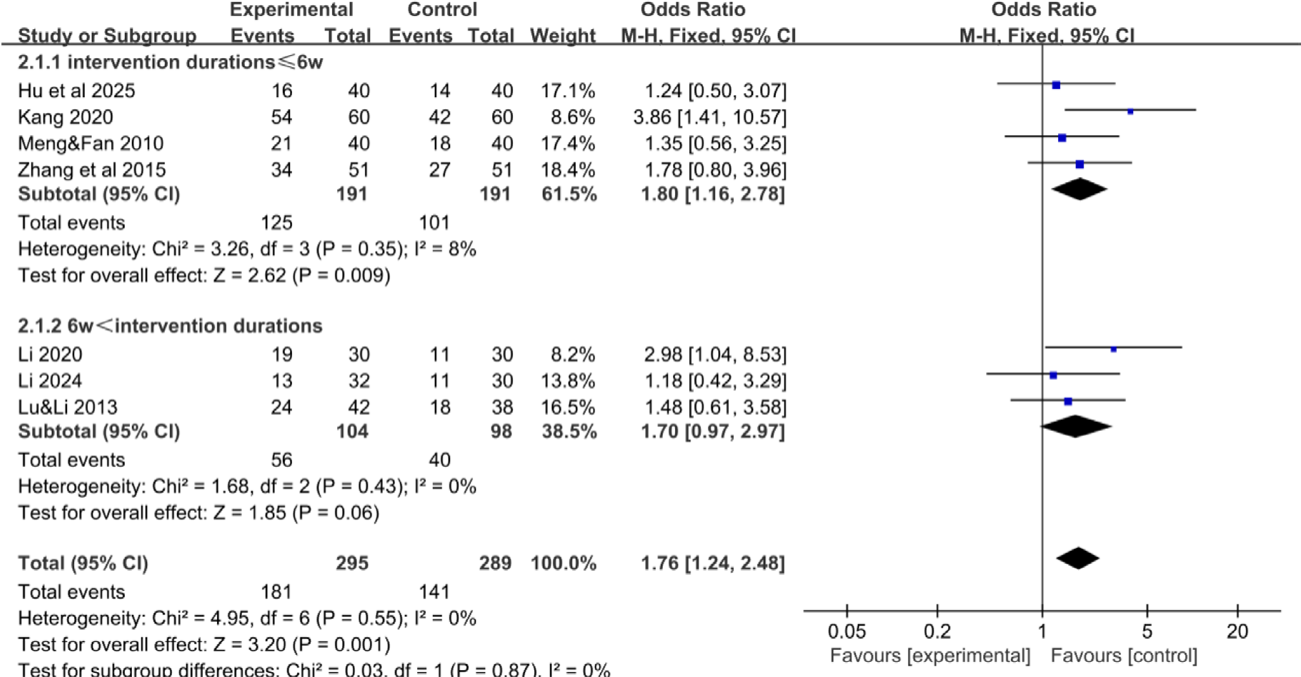
Forest plot of the pooled OR for total effective rate-treatment duration and its 95% CI. CI = confidence interval, OR = odds ratio.

The results indicated that different treatment durations had a certain impact on the total effective rate. The subgroup with a treatment duration of less than or equal to 6 weeks had a higher total effective rate, with a statistically significant difference (*P* < .001), as shown in Figure 11. This finding may be related to the small sample size and limited number of clinical trials. More clinical trials are needed to further validate these results.

#### 3.3.2. Total effective rate-Western medical treatment methods

**Figure 12.**
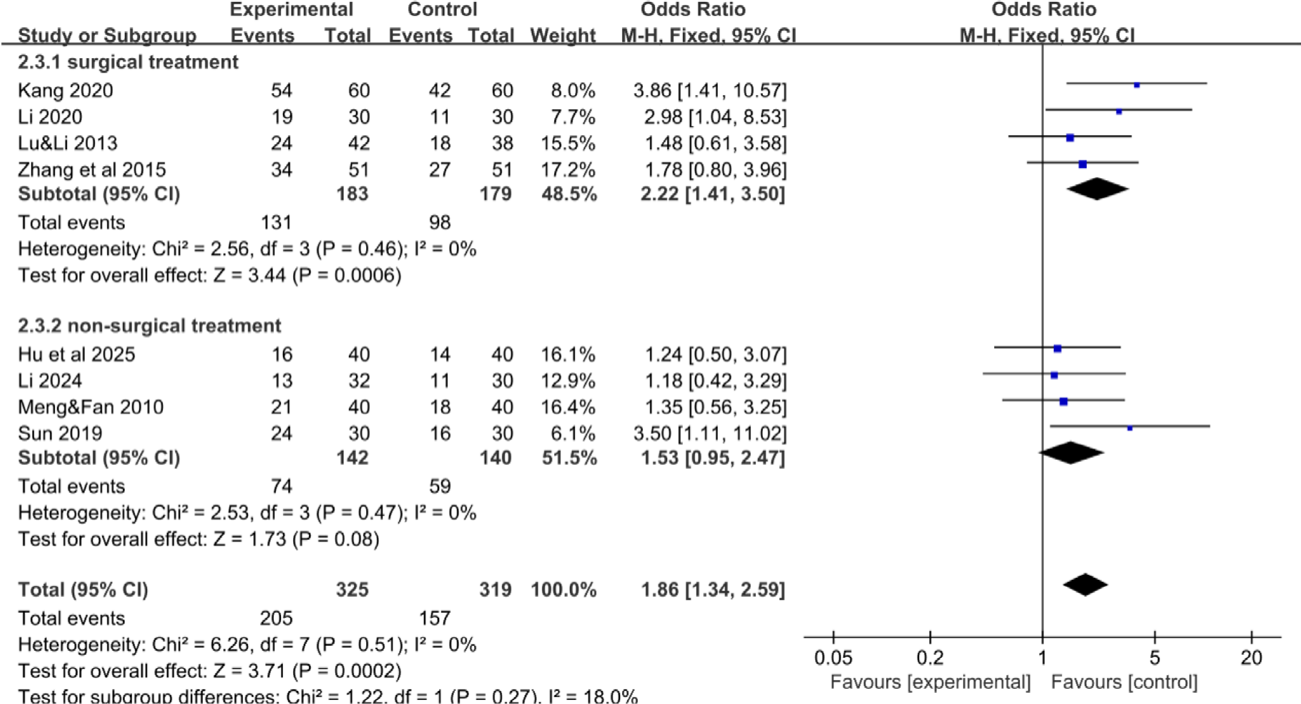
Forest plot of the pooled OR for total effective rate-Western medical treatment methods and its 95% CI. CI = confidence interval, OR = odds ratio.

The results showed that the type of Western medical treatment had a certain impact on the total effective rate. The combination of TCM with nonsurgical Western treatments was superior to the combination with surgical treatments, with a statistically significant difference (*P* < .001), as shown in Figure 12.

#### 3.3.3. KPS score-treatment duration

**Figure 13.**
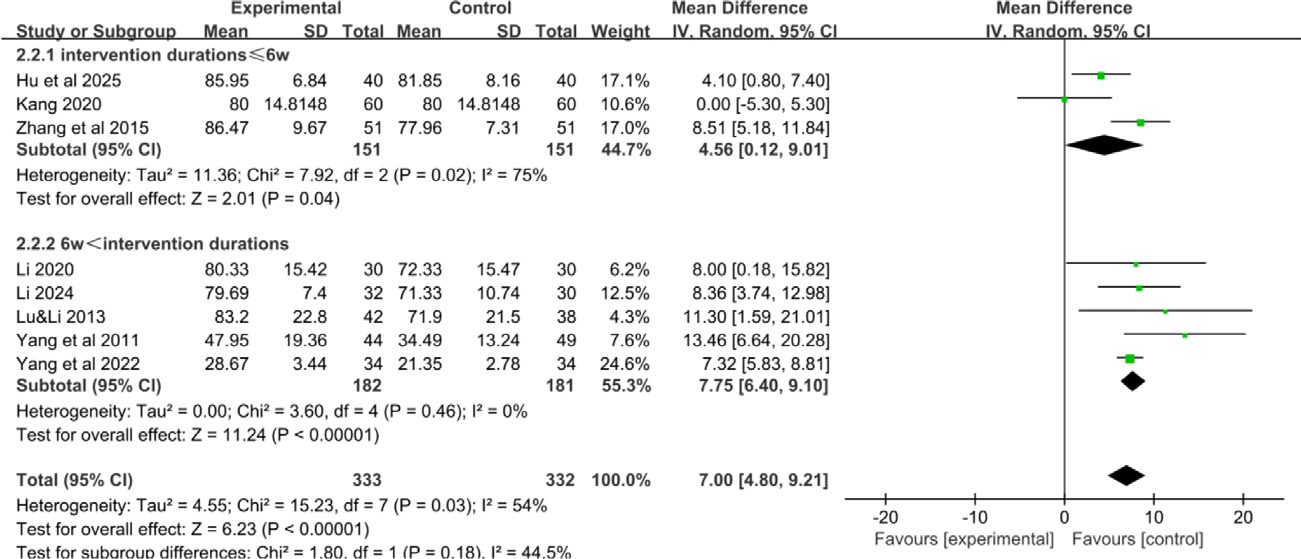
Forest plot of the pooled OR for KPS score-treatment duration and its 95% CI. CI = confidence interval, KPS = Karnofsky performance status, OR = odds ratio.

The results indicated that treatment duration had an impact on the KPS score. The KPS score was higher in the subgroup with a treatment duration of greater than or equal to 6 weeks compared to the subgroup with a treatment duration of <6 weeks, with a statistically significant difference (*P* < .05), as shown in Figure 13.

#### 3.3.4. KPS score-Western medical treatment methods

**Figure 14.**
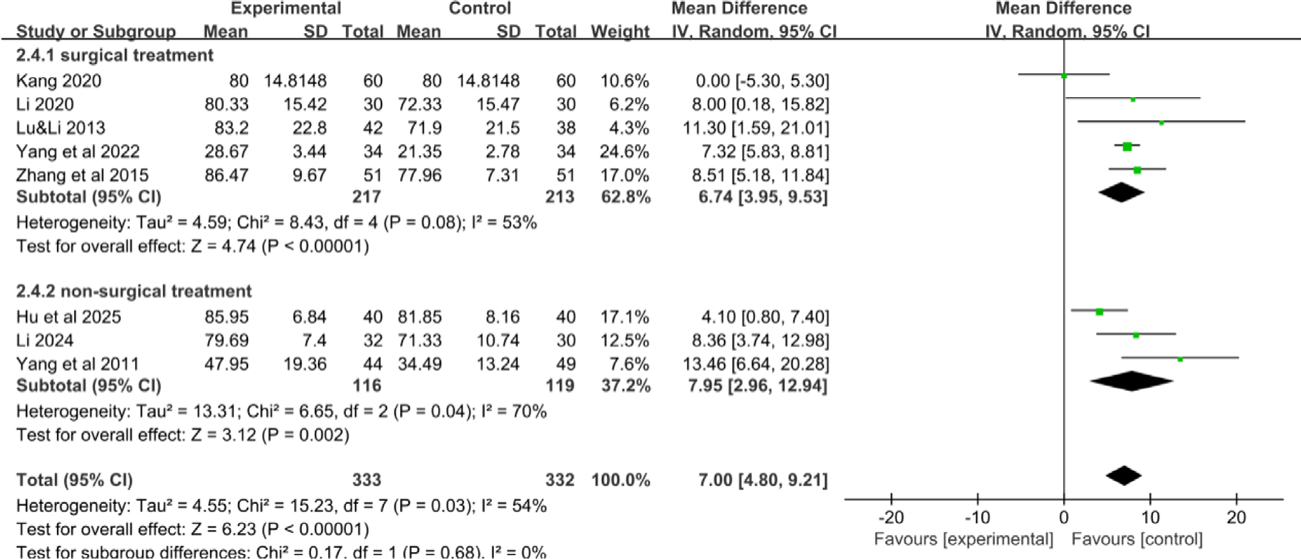
Forest plot of the pooled OR for KPS score-Western medical treatment methods and its 95% CI. CI = confidence interval, KPS = Karnofsky performance status, OR = odds ratio.

The results showed no significant statistical difference between the 2 treatment methods regarding the KPS score (*P* < .01). However, the subgroup combining TCM with nonsurgical Western treatments had higher heterogeneity, as shown in Figure 14.

### 3.4. Sensitivity analysis

A stepwise exclusion method was employed to reassess the total effective rate, KPS score, serum AFP levels, TCM syndrome scores, gastrointestinal adverse reactions, and bone marrow suppression adverse reactions. The results remained largely consistent, with no significant alterations observed. This stability in outcomes suggests that the findings of this study are relatively robust and reliable.

## 4. Discussion

This meta-analysis included 10 RCTs to comprehensively evaluate the clinical efficacy and safety of YGJ in the treatment of PLC. The research results show that the combination of YGJ and Western medicine treatment has significant advantages in improving treatment efficiency, improving patients’ quality of life, reducing tumor marker levels, and alleviating the side effects of Western medicine treatment, providing strong evidence for the application of YGJ in PLC management.

The combination of YGJ and Western medicine significantly improved the treatment efficacy. This indicates that consistent decoction may exert antitumor effects through multiple mechanisms in conjunction with Western medicine treatment.^[27]^ On the 1 hand, various TCM ingredients in the decoction may have direct antitumor activity, the total glucoside of Paeonia lactiflora, a component of YGJ, has been shown to intervene in the PI3K-Akt signaling pathway, thereby reducing the release of serum inflammatory factors IL-6 and TNF-α, while simultaneously increasing the secretion of the anti-inflammatory cytokine interleukin-10 (IL-10).^[28]^ On the other hand, YGJ may also enhance the body’s immune surveillance and clearance ability against tumors by regulating the body’s immune function,^[29]^ thereby improving the therapeutic effect. In addition, the significant improvement in KPS score further confirms the positive effect of YGJ in improving the overall quality of life and functional status of patients. This may be related to the YGJ’s ability to alleviate patients’ symptoms and its positive impact on their psychological state. YGJ can alleviate clinical symptoms such as pain and fatigue in patients, enabling them to better tolerate Western medicine treatment and improve their quality of life.

AFP is a specific biomarker for PLC, and its levels are closely related to tumor burden and treatment efficacy. In this study, YGJ significantly reduced serum AFP levels, this suggests that YGJ may have a positive impact on tumor burden. The TCM ingredients in the decoction may inhibit the growth and proliferation of tumor cells through various pathways, thereby reducing the production of AFP.^[30]^ In addition, YGJ may also improve the metabolic function of the liver and further reduce AFP levels by regulating the internal environment of the body.^[31]^ The decrease in TCM syndrome score also indicates that YGJ has a significant effect in relieving patients’ clinical symptoms. This may be related to the YGJ’s function of nourishing the body and eliminating evil, which improves the patient’s clinical symptoms by regulating the balance of qi, blood, yin and yang in the body.

In terms of safety, YGJ reduced the incidence of gastrointestinal adverse reactions. This indicates that YGJ can alleviate the side effects of Western medicine treatment. This may be related to the consistent spleen and stomach strengthening effects of decoction, which regulate gastrointestinal function and reduce the stimulation of Western medicine treatment on the gastrointestinal tract. However, YGJ did not show significant effects in improving bone marrow suppression. This may be related to the mechanism of action of YGJ. YGJ mainly exerts therapeutic effects by regulating the overall state of the body, while bone marrow suppression is mainly related to the toxicity of Western medicine treatment and may require more targeted treatment measures.

When compared with the meta-analysis that included several articles on the treatment of PLC with TCM,^[13]^ the current study shares the finding that the combination of TCM (YGJ in this case) with western medicine is superior to western medicine alone in improving certain outcomes. Both studies found that the combination treatment could enhance the near-term efficacy of the tumor and improve serum alpha-fetoprotein levels. Additionally, the current study also found improvements in liver function (total bilirubin) and KPS scores, which were not specifically mentioned in the previous meta-analysis.^[14]^ However, the previous meta-analysis reported improvements in immune indexes, which were not evaluated in the current study. While YGJ shares some similarities with other TCM treatments in improving clinical symptoms and enhancing the efficacy of combined treatments, it also has unique aspects, such as its specific impact on liver function and KPS scores. The differences in the evaluation of immune indexes and myelosuppression highlight the need for further research to directly compare the therapeutic efficacy of YGJ with other herbal formulations in the treatment of PLC.

Chinese medicine compound formulas, characterized by their multi-component nature, are capable of exerting multi-level, multi-link integrated regulation. This characteristic is, to some extent, compatible with the complex pathological mechanisms of PLC. Previous research on tumor pathogenesis in TCM has identified proliferating tumor cells as a manifestation of yang, with their occurrence attributed to yin deficiency. Following extensive theoretical, scientific, and clinical analyses, the “Yin Deficiency-Cancer Correlation Hypothesis” was proposed. This hypothesis posits that yin deficiency is the root cause of malignant solid tumors and suggests that nourishing yin can play a role in treating tumors.^[32]^ YGJ, as a representative formula for nourishing yin and soothing the liver, has demonstrated good efficacy when combined with chemotherapy drugs in clinical applications. It not only mitigates the toxic side effects of radiotherapy and chemotherapy but also effectively prevents cancer recurrence and metastasis.^[33]^ Currently, research on YGJ for the treatment of PLC is largely confined to animal experiments or clinical trials. Consequently, this study aims to provide a solid basis for clinical medication by conducting a meta-analysis of clinical studies related to YGJ for PLC.

This study has several limitations: The included studies were limited to Chinese databases, and no relevant literature was found in foreign databases such as PubMed and Web of Science. The consistency of the therapeutic effects of YGJ combined with Western medicine in different regions remains unclear. The studies were geographically restricted to Chinese populations, which may limit the applicability of the results to other ethnic groups. More foreign data need to be included to support the conclusions of this study. None of the included studies employed blinding, and some studies had unclear randomization methods, leading to an overall low quality of the included studies. More high-quality studies are needed to confirm the clinical efficacy of YGJ. The sample size was relatively small, with a total of 745 patients across the 10 included studies. This limits the statistical power and the generalizability of the findings. Since the subgroup analysis results were opposite, further clinical trial data are needed to explore the correlation between YGJ and the total effective rate as well as the KPS score in patients. It is anticipated that future clinical studies on the treatment of PLC with YGJ will involve larger datasets, more rigorous experimental design concepts, and the use of blinding to more objectively collect clinical data, thereby reducing publication bias and enhancing the strength of the research evidence.

## 5. Conclusion

In summary, our findings suggest that combining YGJ or its modifications with Western medical treatments may enhance clinical efficacy, reduce adverse reactions to radiotherapy and chemotherapy, and improve the body’s internal environment. However, these promising results are preliminary and based on limited evidence. Future studies with larger, more diverse samples and rigorous designs are needed to confirm these findings.

## Author contributions

**Conceptualization:** Xindan Cao.

**Data curation:** Zhilin He, Xindan Cao, Li Zhao.

**Formal analysis:** Zhilin He, Xindan Cao, Shidong He.

**Funding acquisition:** Shidong He.

**Methodology:** Zhilin He, Xindan Cao, Li Zhao, Shidong He.

**Project administration:** Xindan Cao.

**Software:** Zhilin He, Xindan Cao, Li Zhao.

**Supervision:** Xindan Cao.

**Validation:** Xindan Cao.

**Visualization:** Xindan Cao.

**Writing – original draft:** Zhilin He, Xindan Cao, Li Zhao, Shidong He.

**Writing – review & editing:** Zhilin He, Xindan Cao, Li Zhao, Shidong He.

## Supplementary Material


