# Supplementary material for: The effectiveness and safety of Yi Guan Jian decoction in the treatment of primary liver cancer: A systematic review and meta-analysis of randomized controlled trials
Source: Medicine (Baltimore). 2026 Jan 9;105(2):e47153. doi: 10.1097/MD.0000000000047153 (PMC12795072; doi:10.1097/MD.0000000000047153)
Supplement: Supplementary file 1 [file medi-105-e47153-s001.docx]

VIP: 18 articles

(M=一贯煎+M=一贯煎加减+M=加味一贯煎+M=一贯煎颗粒) AND (M=原发性肝癌+M=肝癌+M=肝肿瘤+M=晚期肝癌+M=早期肝癌) AND (U=随机)

Wanfang Data: 19 articles

主题 = (一贯煎 OR 一贯煎加减 OR 一贯煎颗粒 OR 加味一贯煎 OR 加减一贯煎) AND 主题 = (肝癌 OR 原发性肝癌 OR 肝肿瘤 OR 早期肝癌 OR 晚期肝癌) AND 全文= 随机

CNKI: 26 articles

(主题：一贯煎 + 一贯煎加减 + 一贯煎加味 + 加味一贯煎 + 一贯煎颗粒) AND (主题：原发性肝癌 + '原发性肝癌(phc)' + '原发性肝癌(plc)' + 肝癌 + 肝肿瘤）AND (全文：随机)

Sinomed: 16 articles：

( "一贯煎"[常用字段:智能] OR "一贯煎加减"[常用字段:智能] OR "一贯煎加味"[常用字段:智能] OR "加味一贯煎"[常用字段:智能] OR "一贯煎颗粒"[常用字段:智能]) AND( "原发性肝癌"[常用字段:智能] OR "肝癌"[常用字段:智能] OR "肝肿瘤"[常用字段:智能]) AND "随机"[全部字段:智能]

Pubmed: 3 articles

检索式：(yiguanjian decoction [Supplementary Concept] OR Yi GuanJian OR Yiguanjian OR Modified Yiguanjian OR Yiguanjian Plus) AND (Primary Liver Cancer OR Liver Cancer OR Hepatic Tumor)

Cochranelibrary: 0 article

#1 (yiguanjian decoction):ti,ab,kw OR (Yi GuanJian):ti,ab,kw OR (Modified Yiguanjian):ti,ab,kw OR (Yiguanjian Plus):ti,ab,kw OR (Yiguanjian):ti,ab,kw

#2 ("primary liver cancer"):ti,ab,kw OR (liver cancer):ti,ab,kw OR (Hepatic Tumor):ti,ab,kw

#1 and #2

Web of science : 0 article

ALL=((yiguanjian decoction OR Yi GuanJian OR Yiguanjian OR Modified Yiguanjian OR Yiguanjian Plus) AND (Primary Liver Cancer OR Liver Cancer OR Hepatic Tumor))
